# Dental Implants Loaded With Bioactive Agents Promote Osseointegration in Osteoporosis: A Review

**DOI:** 10.3389/fbioe.2021.591796

**Published:** 2021-02-09

**Authors:** Cheng Zhang, Tianjia Zhang, Tengyu Geng, Xudong Wang, Kaili Lin, Penglai Wang

**Affiliations:** ^1^School of Stomatology, Xuzhou Medical University, Xuzhou, China; ^2^Affiliated Stomatological Hospital of Xuzhou Medical University, Xuzhou, China; ^3^Department of Oral & Cranio-Maxillofacial Surgery, Shanghai Ninth People’s Hospital, Shanghai Jiao Tong University School of Medicine, Shanghai Key Laboratory of Stomatology, Shanghai Research Institute of Stomatology, Shanghai, China

**Keywords:** dental implants, bioactive agents, osseointegration, osteoporosis, controlled release

## Abstract

Implant-supported dentures are widely used in patients with defect or loss of dentition because these have higher chewing efficiency and do not damage the adjacent teeth compared with fixed or removable denture. An implant-supported denture carries the risk of failure in some systemic diseases, including osteoporosis, because of a non-ideal local microenvironment. Clinically common physical and chemical modifications are used to change the roughness of the implant surface to promote osseointegration, but they have limitations in promoting osteoinduction and inhibiting bone resorption. Recently, many researchers have focused on the study of bioactive modification of implants and have achieved promising results. Herein we have summarized the progress in bioactive modification strategy to promote osseointegration by regulating the local osteoporotic microenvironment.

## Introduction

With innovations in implant design and surgery technology, the applicable conditions of implant surgery have become more extensive, and the 10-year survival rate of an implant-supported denture has exceeded 95% (Buser et al., 2017). There is a risk of failure in a number of systemic diseases (Liu et al., 2020), including osteoporosis, because the severe decrease in bone mass and alteration of trabecular bone microstructure affect the initial stability and osseointegration of the implants.

Currently, the common sand blasting and acid etching strategy is used to increase the surface roughness of implants, which enhances adhesion, proliferation, and differentiation of mesenchymal stem cells (Zhang et al., 2020; Zhao et al., 2020). In addition to the implant design, it is necessary to promote osteoinduction and inhibit bone resorption locally in osteoporotic patients (Lin et al., 2013). Systemic administration orally or intravenously provides low bioavailability, which makes it difficult to maintain an effective concentration around the implants and might cause severe side effects, such as necrosis of the jaw bone (Eguia et al., 2020). Local administration ensures adequate drug concentration in the target tissue and reduces toxicity in the non-target areas. Hence, it is necessary to biologically modify the implants for loading bioactive agents, such as anti-osteoporosis drugs, bioactive molecules, or bioactive inorganic elements, onto the implant surface. In addition, to avoid burst release in a short time, it is critical to take optimal approaches to optimize the locally controlled release of bioactive agents and maintain an effective concentration. Herein we briefly summarize the main progress in this field.

## Loading Bioactive Agents to Promote Osseointegration

### Loading Anti-osteoporosis Drugs

**FIGURE 1:**
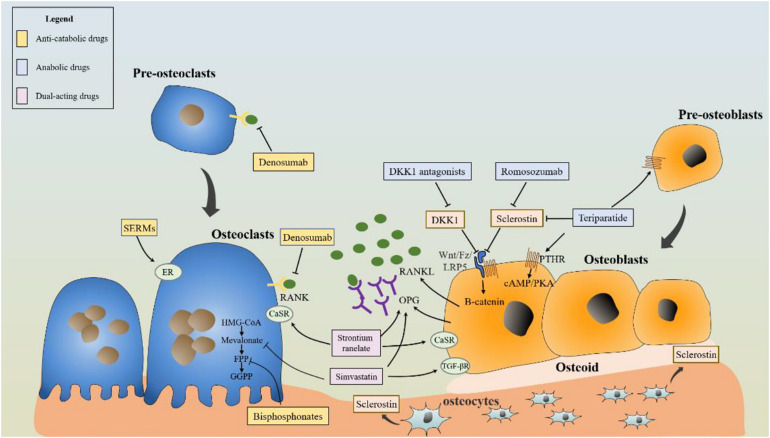
Schematic diagram of anti-osteoporosis drugs acting on osteoblasts and osteoclasts.

Anti-osteoporosis drugs are categorized according to their functions and effects and include anti-catabolic drugs, anabolic drugs, and dual-acting drugs (Figure 1). The commonly used anti-catabolic agents for loading on the implant surfaces for local treatment include bisphosphonates, receptor activator of nuclear factor kB ligand (RANKL) antibodies, and selective estrogen receptor modulators (SERMs), which inhibit the activity and recruitment of osteoclasts or promote the apoptosis of osteoclasts. Bisphosphonates reduce osteoclast activity by inhibiting farnesyl pyrophosphate synthase and protein prenylation in the mevalonate pathway, and SERMs have an agonist effect on the estrogen receptor in osteoclasts, thereby inducing apoptosis (Apostu et al., 2017). Beck et al. (2019) immobilized the RANKL antibody denosumab on the implant surface to inhibit osteoclast differentiation, which could competitively block the interaction between receptor activator of nuclear factor kB and RANKL.

Being an anabolic drug, the parathyroid hormone (PTH) activates the cyclic adenosine monophosphate/protein kinase A (cAMP/PKA) signaling pathway by acting on the PTH receptors in osteoblasts to promote osteointegration (Tang et al., 2020). PTH 1–34 or teriparatide, a fragment of endogenous PTH, was the main regulator of calcium and phosphate metabolism in bone and the first anabolic drug proven to increase the osseointegration of implants. Other anabolic drugs targeting the Wnt/β-catenin signaling pathway have also been studied, such as romosozumab and DKK1 antagonists (Gennari et al., 2020).

Strontium ranelate and simvastatin play dual effects of promoting bone formation and inhibiting bone absorption through various signaling pathways (Apostu et al., 2017). Loading simvastatin and strontium ranelate on the implant surface effectively improves the local bone microenvironment, which is a promising method to enhance the osseointegration of implants in osteoporotic patients (Lai et al., 2018).

### Loading Bioactive Molecules

The term “bioactive agents” is not limited only to therapeutic agents used in the clinic. The scope of this term has been broadened to bioactive molecules, including growth factors, proteins, and genes (Meng et al., 2016; Souza et al., 2019). Although the clinical application of these bioactive molecules is limited because of high production costs and concerns about biosafety, researchers have conducted a lot of research in this area. Loading various growth factors and proteins on the implant surfaces can promote osteogenic differentiation and the mineralization of bone marrow stem cells. Platelet-derived growth factor, insulin-like growth factor, fibroblast growth factor, vascular endothelial growth factor (VEGF), and bone morphogenetic protein (BMP) have been widely used in this field (Chen and Zou, 2019; Jurczak et al., 2020). VEGF can promote angiogenesis and regulate bone regeneration; Zavan et al. (2017) loaded VEGF on the surface of the implant *in vitro*, which effectively enhanced the osteogenic differentiation of stem cells. The release of BMP from the implant surface facilitated the proliferation, differentiation, and mineralization of bone cells *in vitro* and enhanced bone healing *in vivo* (Teng et al., 2019).

In addition, the extracellular matrix, such as type I collagen, showed good biological activity and osteoinductivity, which could improve adhesion and differentiation and promote bone-to-implant integration (Wang et al., 2019). Similarly, genes can be incorporated into the implant surface to transfect local osteoblasts or osteoclasts around the implants to promote osteoblastogenesis and inhibit osteoclastogenesis. Takanche et al. (2018) performed an experiment in which c-myb, a transcription factor, was delivered from chitosan-gold nanoparticle-coated titanium surface to the target tissue, where it promoted bone formation under osteoporotic conditions.

### Modification by Bioactive Inorganic Elements

Some essential elements, including calcium (Ca), strontium (Sr), magnesium (Mg), zinc (Zn), and silicon (Si), can be also loaded on implants to stimulate osteogenesis (Lin et al., 2013; Liu et al., 2020, 2021). Compared with anti-osteoporosis drugs and bioactive molecules, more efficient strategies can be applied to construct inorganic element coating on the implant surface, which costs less (Asri et al., 2017). Ca is one of the essential micronutrients in bone, and bone structure abnormalities in osteoporotic patients occur as a result of the loss of Ca. At present, Ca-phosphate biomimetic coating on the implant surface is widely used to promote adhesion and differentiation of osteoblasts because of the chemical similarity between the synthetic materials and the bone mineral components (Xia et al., 2018). Sr is often used in the treatment of osteoporosis because of its dual role in bone regulation. An Sr-incorporated implant surface obtained by hydrothermal reaction could promote early osseointegration in osteoporotic rabbits (Lin et al., 2019). Moreover, Mg-immersed titanium-dioxide (TiO_2_) coatings on the implant surface had both osteogenic and antibacterial effects (Zhao et al., 2019). Zn-modified coating on the implant surface facilitated the osteogenic differentiation of bone marrow-derived pericytes through the transforming growth factor-beta/Smad signaling pathway (Yu et al., 2017).

## Controlling the Release Behaviors of Bioactive Agents

There are many ways to build bioactive organic or inorganic coatings on the implant surface, including physical methods (such as plasma spraying, ion implantation, and physical vapor deposition), chemical methods (such as acid etching and alkali-heat treatment), and electrochemical strategies (such as anodization, micro-arc oxidation, electropolymerization, and electrophoretic deposition) (Asri et al., 2017; Xue et al., 2020). Covalent grafting and layer-by-layer self-assembly technology have been used to load bioactive proteins and growth factors. The release of bioactive agents from the implant surface is different because of the different construction methods of the coatings. In practical applications, however, we expect that the drugs loaded on the implants should maintain an effective concentration locally for a long time because burst release not only fails to maintain long-term efficacy but also might cause side effects due to toxicity in local tissues. The controlled release behaviors of the loaded bioactive agents can be achieved mainly by the following means: (1) constructing micro-/nano-structures on the implant surface, (2) introducing a stable immobilization strategy, and (3) encapsulating the bioactive agents.

### Constructing Micro-/Nano-structures on the Implant Surface

**FIGURE 2:**
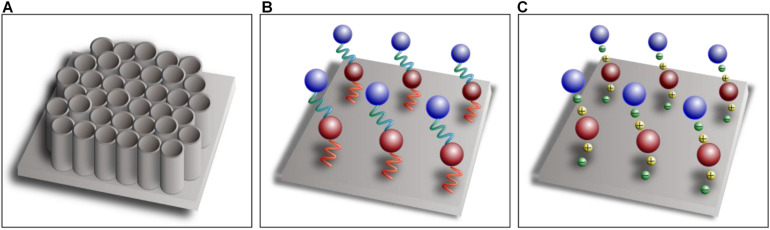
Schematic diagram of titanium dioxide nanotubes **(A)**, covalent immobilization **(B)**, and layer-by-layer self-assembly technique **(C)**.

Nano- and micro-topography construction on the implant surface not only can provide better biological responses but also may benefit drug adhesion and controlled release (Wang et al., 2018, 2020; Long et al., 2019). The modification of titanium dioxide nanotubes (TNTs) on implant surfaces can significantly increase the surface-to-volume ratio, and this porous surface provides more substantial space for drug loading with better biocompatibility (Figure 2A). More importantly, bioactive agents incorporated on the surface and inside TNTs achieve sustained steady release (Ion et al., 2020). A study conducted by Liu et al. (2018) showed that zoledronate adsorbed on the surface of TNTs can be released steadily for a long time to enhance implant osseointegration. Moreover, the drug release behaviors can be regulated by the diameter and length of TNTs by varying the process conditions of anodic oxidation. Hamlekhan et al. (2015) found that the diameter, length, aspect ratio, and volume were related to the prolonged release process. Moreover, the aspect ratio had the highest correlation with the release rate, and the release process of TNTs with high aspect ratio was significantly slow.

### Introducing a Stable Immobilization Strategy

Bioactive agents can be attached to the implant surface by means of physical adsorption, such as dipping, spray coating, or drop casting, to promote local osseointegration (Alenezi et al., 2019). Physical adsorption characterized by Van der Waals forces or hydrogen bonds, however, leads to burst release at an early stage. Hence, bioactive agents can be covalently immobilized on the surface of titanium indirectly through a separate linker molecule that mediates the binding between titanium substrates and bioactive agents (Figure 2B), which can greatly improve the stability of coatings compared with physical adsorption (Jin et al., 2020). Linker molecules are generally synthetic linkers, such as silane and polyethylene glycol, or biologically derived molecules, such as heparin, dopamine, and chitosan. Linker molecules attach to hydroxyl-functionalized titanium substrates through condensation reactions, and then the bioactive agents are covalently immobilized on the functional group of the linker molecules (Stewart et al., 2019). Covalent bonding is more complex and time consuming than the other techniques, however, and the bioactive agents are not easily released because of the tight covalent bonding (Ma et al., 2020). The layer-by-layer self-assembly technique is being increasingly used for drug loading and controlled release, which can formulate polyelectrolyte multilayers by electrostatic attractions between components with different electric charges (Figure 2C). The self-assembly process is simple and mild, which does not affect the activity of the components, and sustained-release administration can be achieved by adjusting the physical and chemical properties of the materials (Shi et al., 2017).

### Encapsulating the Bioactive Agents

**FIGURE 3:**
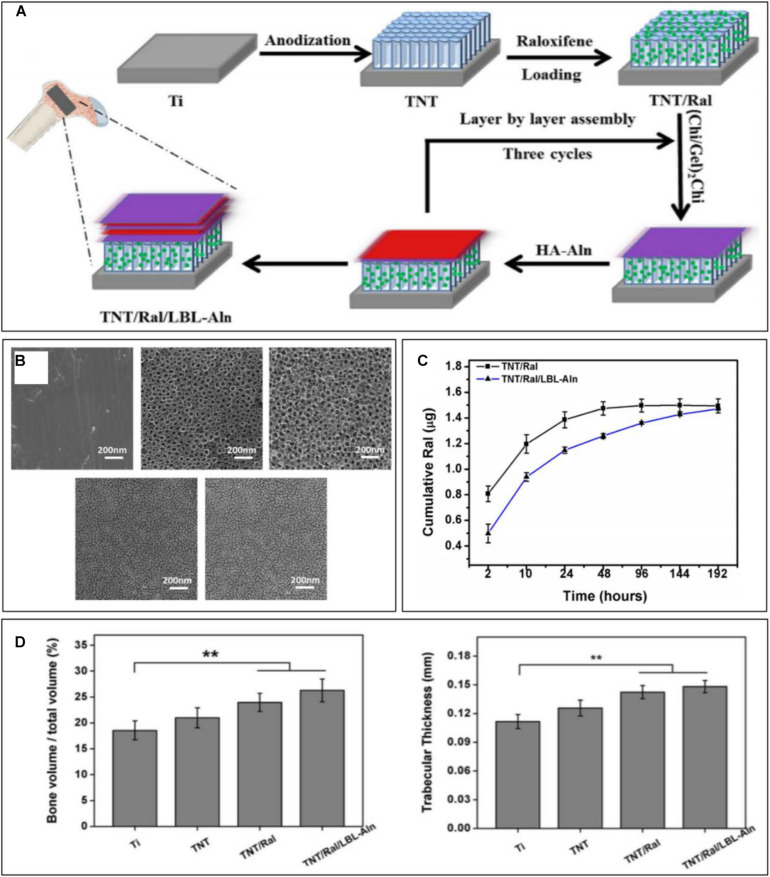
Sustained raloxifene release from TiO_2_ nanotubes to enhance osteointegration. **(A)** Schematic illustration of the fabrication of multilayered coating. **(B)** SEM images of each group. **(C)** Cumulative release curve of each sample. **(D)** Quantitative analysis of new bone volume and trabecular thickness [reprinted with permission from Mu et al. (2018); copyright (2018) Elsevier]. “**” Means Statistically significant differences are denoted by symbols, **p < 0.01.

Bioactive agents can be encapsulated by biocompatible materials with appropriate biodegradability, including natural organic polymers [such as chitosan (CS) and gelatin], synthetic organic polymers [such as hyaluronic acid (HA) and polycaprolactone (PCL)], and inorganic materials (such as calcium phosphate). With continuous degradation of the encapsulation materials, the bioactive agents are gradually released into the target area to achieve sustained release (Figure 3). To locally release microRNA-21, Wang et al. (2015) used CS and HA to encapsulate miRNA-21 to fabricate the CS/HA/miRNA-21 nanoparticles, which were then crosslinked with gelatin and loaded onto the implant surface, ultimately promoting the expression of the osteogenic gene. PCL and poly(lactic-co-glycolic) acid can also be used as encapsulation materials of bioactive agents to achieve local controlled release (Littuma et al., 2020). Biomimetic coprecipitation is a method of encapsulating bioactive agents with inorganic materials. With absorption of the hard coating, the bioactive agents are released gradually.

## Conclusion and Perspective

Herein we have summarized the bioactive agents available to improve osseointegration in osteoporotic patients and the methods of controlled release of bioactive agents. High bioavailability and low toxicity in tissues outside the target make biomodification of implants suitable for achieving local osseointegration. Concerns about biosafety, however, limit the popularization of bioactive modification of implants, which is the direction of follow-up research. Implant osseointegration also must be accompanied by angiogenesis and ingrowth, and it is an inflammatory regulatory process, which is initially mediated by M1 phenotype macrophages and subsequently by M2. In addition, bacterial colonization and formation of plaque biofilms on the surface of implants may cause peri-implantitis, ultimately leading to failure of the implant. Therefore, angiogenesis and ingrowth, regulation of the inflammatory response, and inhibition of biofilm formation also should be considered in future studies.

## Author Contributions

CZ and TZ wrote the original draft equally. TG helped to prepare the manuscript. XW gave conceptualization on this manuscript. KL and PW led the conceptualization and project administration, and supervised the writing and editing of the manuscript. All authors contributed to the article and approved the submitted version.

## Conflict of Interest

The authors declare that the research was conducted in the absence of any commercial or financial relationships that could be construed as a potential conflict of interest.
